# Immunomodulation Induced by Host Pathogen Interaction

**DOI:** 10.1155/2019/9710910

**Published:** 2019-08-29

**Authors:** Hadi M. Yassine, Syed M. Moin, Farhan S. Cyprian, Adam K. Wheatley, Gheyath K. Nasrallah

**Affiliations:** ^1^Biomedical Research Center, Qatar University, Doha 2713, Qatar; ^2^College of Health Sciences, QU Health, Qatar University, Doha 2713, Qatar; ^3^Vaccine Research Center, NIAID, NIH, Bethesda MD, USA; ^4^College of Medicine, QU Health, Qatar University, Doha 2713, Qatar; ^5^Department of Microbiology and Immunology, University of Melbourne, Parkville, Australia

Controlling and preventing infections require deep understanding of the complex interplay that occurs between the host and pathogen following infection. In essence, immunomodulation is any process leading to an immune response that can be altered to a desired level. In mammals, the immune system has developed an extensive array of cells and immunomodulators to recognize, identify, and eliminate foreign invaders. On the other hand, pathogens have evolved multiple mechanisms to combat the host immune system as they establish infections. In this context and under certain circumstances, an infection may result in a subverted immune system, which may lead to an exacerbated illness. Recent advances in biotechnology have enhanced our knowledge of the complex interplay that occurs between the host and invading pathogens following infection, through understanding of the microbial virulence strategies as well as the host's approaches to combat the infection.

This special issue collects six high-quality papers, four research articles and two reviews, related to understanding immunomodulation in the context of viral and parasitic infections.

In the article titled “*Taenia crassiceps*-Excreted/Secreted Products Induce a Defined MicroRNA Profile that Modulates Inflammatory Properties of Macrophages” by D. Martínez-Saucedo et al., the authors described how *Taenia crassiceps*-excreted/secreted antigens (TcES) can modulate proinflammatory responses in macrophages by inducing regulatory posttranscriptional mechanisms and, hence, reduce detrimental outcomes in hosts running with inflammatory diseases. In summary, their study demonstrates a role for TcES in regulating the production of key inflammatory cytokines, “possibly by inducing microRNAs that target inflammatory transcripts and promoting the release of IL-10 in macrophages.” This phenomenon shapes the transcriptomic profile of macrophages and consequently the outcome of the immune response. These findings increase the basic understanding of how the released molecules from helminths would regulate inflammation and, thus, may offer new approaches for the treatment of autoimmune and inflammatory diseases.

The remaining three research articles focused on viral infections of three different families, namely, the lymphocytic choriomeningitis virus (LCMV), human papillomavirus type 16, and MERS-coronavirus. The first of these studies was titled “Lymphocytic Choriomeningitis Virus Infection Demonstrates Higher Replicative Capacity and Decreased Antiviral Response in the First-Trimester Placenta” by E. A. L. Enninga and R. N. Theiler from the University of Texas Medical Branch. LCMV is a zoonotic pathogen, of which rodents are the natural reservoir. Although the majority of persons infected with LCMV show relatively minor illness, nonetheless, the virus can cross the placental barrier during pregnancy and cause congenital defects in fetuses. In this study, differences in immunomodulation between the first- and third-trimester placental explants infected with LCMV were evaluated. Generated data suggested that the innate immune response to LCMV infection of the human placenta is more vigorous in the third trimester compared to the first trimester. The authors attributed the absence of viral replication in term placental explants to the robust innate antiviral response in this tissue. These findings are in agreement with clinical observations of decreased transplacental transmission and less severe fetal phenotypes of viral pathogens acquired in later gestation.

In the other study titled “Human Papillomavirus Type 16 Disables the Increased Natural Killer Cells in Early Lesions of the Cervix,” J. Zhang et al. retrospectively analyzed the histologic pathology results of 245 women with HPV type 16 only (HPV16+) or type 18 only (HPV18+). The study was the first on emphasizing the unique immune profiles of the cervical microenvironment between two high-risk HPV types. In summary, the authors indicated that more severe lesions are found in the cervix of HPV16+ women compared with those in HPV18+ women. Briefly, their data demonstrated that the number of NK cells was increased but their cytotoxic function was abnormal in an HPV16-infected cervix. The involved mechanisms may partially explain why HPV16 is the most likely to cause cervical cancer and may provide new potential strategies for its clinical management.

Several serological studies indicated that the Middle East respiratory syndrome coronavirus (MERS-CoV) has been circulating in camels in the Arabian Peninsula for more than two decades. However, it is still intriguing why the virus was first detected in humans in 2012. It is worth noting that infection with MERS-CoV could be asymptomatic or cause mild influenza-like illness. This may suggest that the prevalence of MERS-CoV infections in the general population is underestimated. The aim of the study by R. A. Al Kahlout et al. was to evaluate the performance of various serological assays and to estimate the seroprevalence of anti-MERS-CoV antibodies in high- and low-risk groups in Qatar. The paper reported low prevalence of anti-MERS antibodies in the general population, which coincides with the low number of all reported cases by the time of their study. Importantly, serological analysis indicated high cross-reactivity between MERS-CoV and other coronaviruses, which necessities more detailed investigation of the immune responses to coronavirus infections.

One of the two reviews in this issue by W. Zeng et al. examined the transplantation of probiotics and fecal microbiota on immunomodulation. Probiotics or microbiota are commensal/nonpathogenic microbes that provide beneficial effects to the host through several mechanisms, including but not limited to competitive exclusion of pathogenic bacteria and modulation of immune responses. The aim of this review was to follow through the recent literature on immunomodulatory effects and mechanisms of probiotics and fecal microbiota transplantation (FMT) and also the efficacy and safety of probiotics and FMT in clinical trials and applications. The authors concluded that the immunomodulation induced by probiotics is a complex interplay between different hosts and microorganisms, and hence, the immunomodulatory characteristics of specific probiotics cannot be generalized. Accordingly, “personalized probiotics intervention and standardized fecal bacteria transplantation should be challenges and prospects for future research on the intervention model of intestinal flora.”

Macrophages are indispensable modulators of the innate immune system because they maintain a delicate balance between immunity against pathogenic bacteria and tolerance of commensals in the intestine. In their review titled “Functions of Macrophages in the Maintenance of Intestinal Homeostasis,” S. Wang et al. focused their attention on intestinal macrophages, “describing the recent insights into the role of intestinal macrophages in maintaining gut homeostasis and managing gut inflammation.” They also discussed the nutritional modulation of intestinal macrophage functions and the potential of nutritional strategies to manipulate intestinal macrophages and ameliorate inflammatory bowel disorders. They concluded that “a better understanding of mechanisms employed by intestinal macrophages in maintaining intestinal homeostasis and the action of enteral nutrients in the regulation of intestinal macrophages will facilitate the development of nutritional strategies in gut health improvement as well as prevention and control of inflammatory bowel disorders.”

This special issue provides original research and review articles that would be helpful to experts and broad audience alike in enhancing their understanding of the complex host-pathogen interactions.

